# Influence of the Transducer-Mounting Method on the Radiation Performance of Acoustic Sources Used in Monopole Acoustic Logging While Drilling

**DOI:** 10.3390/s25010201

**Published:** 2025-01-01

**Authors:** Jiale Wang, Xiaohua Che, Wenxiao Qiao, Shengyue Tao, Qiqi Zhao

**Affiliations:** 1 State Key Laboratory of Petroleum Resources and Prospecting, China University of Petroleum, Beijing 102249, China; 2020310408@student.cup.edu.cn (J.W.); qiaowx@cup.edu.cn (W.Q.); taosy97@foxmail.com (S.T.); zhaoqiqihitwh@163.com (Q.Z.); 2 Key Laboratory of Earth Prospecting and Information Technology, Beijing 102249, China

**Keywords:** acoustic logging while drilling, mounting method, radiation performance, harmonic-response analysis, transient response analysis

## Abstract

Transducers used in acoustic logging while drilling (ALWD) must be mounted on a drill collar, and their radiation performance is dependent on the employed mounting method. Herein, the complex transmitting voltage response of a while-drilling (WD) monopole acoustic source was calculated through finite-element harmonic-response analysis. Subsequently, the acoustic pressure waveform radiated by the source driven by a half-sine excitation voltage signal was calculated using the complex transmitting voltage response. The calculation results were compared with those obtained using finite-element transient analysis to verify the accuracy of the calculation method. The influence of transducer-mounting methods on the radiation performance of the monopole acoustic source was examined by modifying the material and structural dimensions of the coupling medium between the transducer and drill collar as well as the material and thickness of the protective cover. Numerical simulations were performed, and a transducer-mounting method suitable for ALWD was proposed based on the simulation results. Results showed that soft rubber (as the coupling material; thickness = 2 mm) enabled the WD monopole acoustic source to radiate robust acoustic energy in an infinite fluid. Increasing the height of the coupling material enhanced the radiated acoustic energy and reduced axial vibrations on the drill collar. The radiated acoustic pressure signal was unaffected by a steel protective cover (thickness = 0.5 mm). Conversely, increasing the cover thickness reduced the energy of the radiated acoustic signal. With increasing pulse width of the half-sine excitation voltage signal, the amplitude of the radiated acoustic pressure of the transducer initially increased and then declined, reaching a maximum at a pulse width that was 0.6 times the resonant period. Overall, the findings help in designing acoustic-source structures and excitation signals for ALWD tools.

## 1. Introduction

Acoustic logging (AL) technology can be employed to determine the elastic properties, mechanical properties, and reservoir characteristics of formations; it can also be used to evaluate the quality of well cementing. In addition, it is widely used in the exploration and development of oilfields. As an advanced form of this technology, acoustic logging while drilling (ALWD) allows logging operations to be conducted while drilling (WD). Compared with traditional wireline logging, ALWD substantially reduces overall costs but faces challenges, such as drill collar waves, drilling noise, and mudflow noise interference [1,2,3]. The characteristics of acoustic transducers influence the quality of ALWD data. To improve the signal-to-noise ratio and detection depth of AL instruments, the transmitting transducer must have a high transmitting voltage response [4,5].

The ALWD tool is an acoustic measurement system based on a thick-walled metal tube structure (drill collar). The design of the WD transmitting transducer is constrained by the specific measurement conditions that it must meet. The transducer is typically mounted on a slotted drill collar. To facilitate installation, piezoelectric vibrators are frequently designed to have an arc shape. The arc-shaped piezoelectric vibrator must be encapsulated to provide electrical insulation and enhance the resilience of the transducer to fluid erosion [6,7]. This type of transmitting transducer is common in the ALWD tools used by numerous oil service companies. Examples include Baker Hughes’ APX and Schlumberger’s Sonic Scope, which employ eight and four arc-shaped piezoelectric vibrators, respectively, to generate the WD acoustic source [8,9]. To optimize the radiation performance of the acoustic source, it is essential to enhance the structural design of the vibrators. The most prevalent numerical simulation approach is the finite-element method (FEM), which considers the structural characteristics of the transducer and the associated electromechanical coupling effects, as well as the coupling between the transducer and surrounding medium [10,11]. The effects of geometry and boundary conditions on the radiation performance of a WD-transmitting transducer have been analyzed using the FEM [12,13,14]. The objective of such numerical simulation studies was to provide a theoretical basis for the dimensional design of arc-shaped piezoelectric vibrators. However, only a few studies have considered the impact of the encapsulated structure of the vibrator on the radiation performance of the transducer [13,15]. In addition to the encapsulated structure, the acoustic performance of the transducer can be affected by the mounting method. This encompasses the coupling material between the drill collar and transducer, the structure of the drill collar, and the protective cover, which influence the radiation characteristics of the WD transmitting transducer, constituting a gap in the existing literature.

This paper presents an analysis of the transmitting voltage response of the WD monopole acoustic source in the frequency domain, employing the multiphysical field FEM. The radiated acoustic pressure waveforms generated by the WD monopole acoustic source under the excitation of the half-sine excitation voltage signal and the axial vibration velocity waveforms on the drill collar surface were calculated based on the frequency response. The impact of the transducer-mounting method on the radiation performance of the WD monopole acoustic source was investigated by modifying the material properties, thickness, and height of the coupling material, as well as the material and thickness of the protective cover. Based on the study findings, we proposed a mounting method for the WD monopole acoustic source. The proposed method enables the WD monopole acoustic source to radiate strong acoustic pressure waveforms with weak axial vibration excitation on the drill collar.

## 2. Materials and Methods

### 2.1. Calculation Model

**Figure 1:**
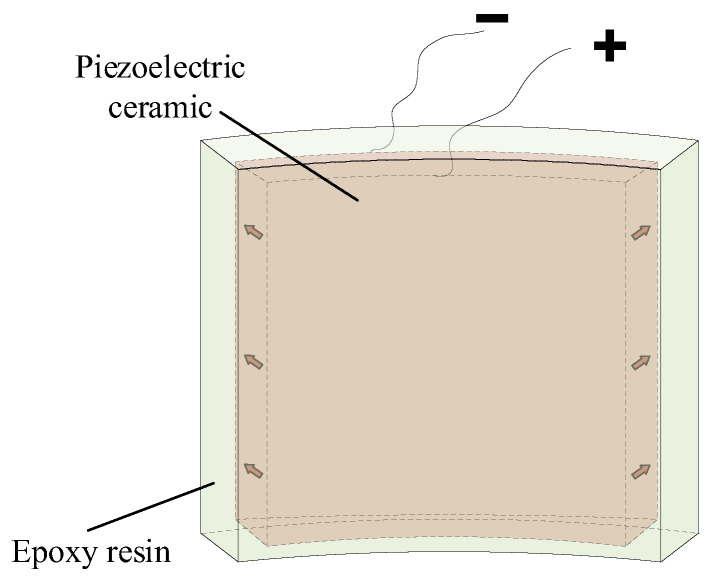
Schematic structure of an arc-shaped piezoelectric vibrator after epoxy resin encapsulation.

Piezoelectric ceramic materials are generally used in transmitting transducers in ALWD tools to achieve energy conversion between electricity and sound. Such transducers generally comprise four arc-shaped piezoelectric vibrators, and each vibrator must be encapsulated in epoxy resin or rubber. Figure 1 shows the configuration of an arc-shaped piezoelectric vibrator after epoxy encapsulation. The polarization of the arc-shaped piezoelectric ceramic was in the radial direction, and the material was PZT-5G. The encapsulated vibrator exhibited a resonance frequency of *f*_0_ = 15 kHz under the condition of mechanical freedom in a fluid.

**Figure 2:**
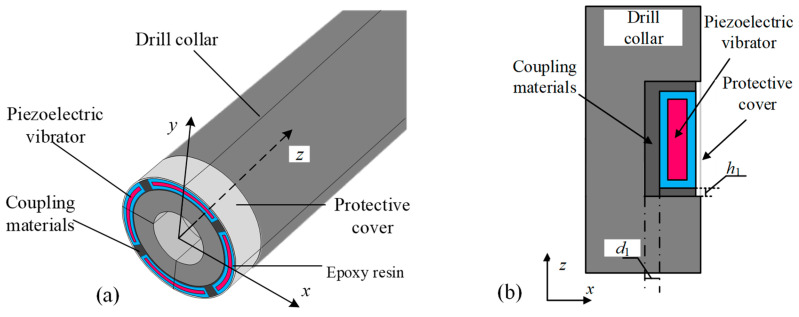
Three-dimensional structure diagrams (**a**) and cross-sections (**b**) of a while-drilling monopole acoustic source.

The transmitting transducers for downhole applications require a high transmitting voltage response and environmental stability against high temperature, high pressure, vibration, and fluid erosion. The conventional mounting approach to cable AL, which involves directly inserting transmitting transducers into silicone oil-filled rubber bladders, is no longer compatible with ALWD. Figure 2 shows the schematic structure of the WD monopole acoustic source. The axial distances between the upper and lower edges of the encapsulated arc-shaped piezoelectric vibrator and the boundary of the drilling collar notch in the cross-section shown in Figure 2b were both designated as *h*_1_. In addition, there was a radial width of the gap between the inner arc of the vibrator and the drilling collar, which is labeled as *d*_1_. To ensure the stability of the transducer under drilling conditions and to extend the service life of the transducer, it is necessary to fill the gap between the drill collar and encapsulated vibrator with coupling material WD, and the structure of the coupling material changes with a change in the structure of the groove engraved in the drill collar. Furthermore, a protective cover must be applied at the outermost layer to provide additional protection for the transducer. Table 1 and Table 2 show the elastic parameters of each medium in the calculation model and the material parameters of PZT-5G, respectively.

### 2.2. Calculation Method

#### 2.2.1. Complex Transmitting Voltage Response Calculations

The complex transmitting voltage response (*S_v_*) is used to describe the relation between the voltage applied to the transmitting transducers and the radiated acoustic pressure signal. When subjected to harmonic excitation at a specific frequency, the *S_v_* (unit: Pa·m/V) is defined as the acoustic pressure *p*_0_ (unit: Pa) at a certain reference distance, *d*_0_ (unit: m) from the acoustic center in a specified direction, multiplied by the reference distance *d*_0_ and then divided by the input voltage *u*. This can be expressed as follows:

(1) $$S_{v}=p_{0}d_{0}/u.$$

The magnitude and phase of the *S_v_* were defined as follows:

(2) $$\left|S_{v}\right|=\sqrt{Re{\left(S_{v}\right)}^{2}+Im{\left(S_{v}\right)}^{2},}$$

(3) $$Phase\left(S_{v}\right)=arctan\left(\frac{Im\left(S_{v}\right)}{Re\left(S_{v}\right)}\right).$$

**Figure 3:**
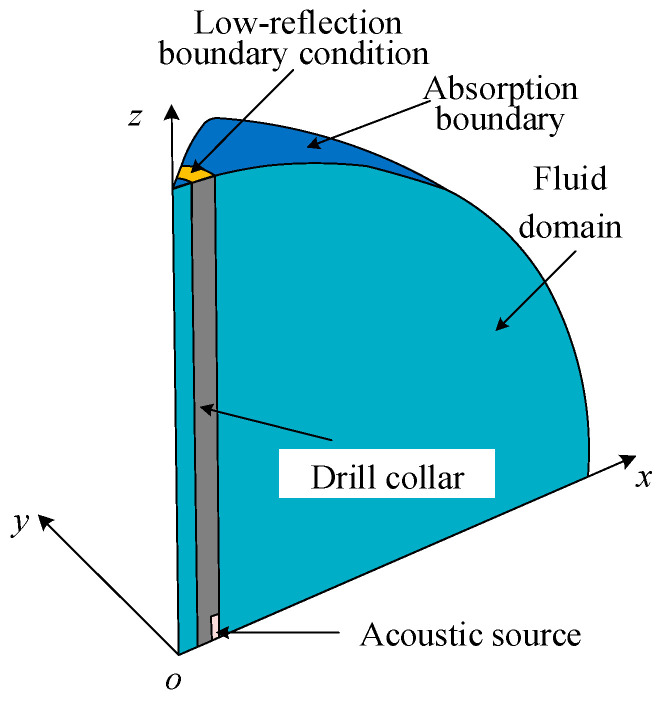
Computational model for the analysis of the harmonic response of a monopole acoustic source in an infinitely large fluid (1/16 computational model).

For the complex structure of the transducers, theoretically deriving the formula of its transmitting voltage response is impossible, and the FEM is a numerical calculation method that is currently used widely in the engineering field. Furthermore, the FEM can be employed to solve almost all engineering problems. Herein, the computational model of the finite-element harmonic-response analysis of a WD monopole acoustic source is established in a three-dimensional Cartesian coordinate system, as shown in Figure 3. When the acoustic source functions in the monopole mode, the excitation voltage signals applied to the four arc-shaped piezoelectric vibrators uniformly arranged in the circumferential direction are the same, as well as the vibration amplitude and phase of each vibrator. Thus, to improve the calculation efficiency, the calculation area was set to 1/16 of the spherical fluid domain according to the symmetry of the model. The radius *R* of the fluid domain should satisfy the far-field calculation conditions, as follows:

(4) $$R\geq \pi \frac{D^{2}}{\lambda }=\pi \frac{D^{2}f}{c},$$

where *D* is the maximum linear dimension of the transducer (the *D* in Figure 1 is 0.11 m), *λ* is the wavelength of the acoustic wave in the fluid, and *c* is the propagation velocity of the acoustic wave in the fluid. The frequency range of the signals commonly used for AL is generally <20 kHz. In this study, the density of the fluid medium was 1000 kg/m^3^, and the acoustic velocity was 1500 m/s; thus, the radius of the fluid domain calculation, *R*, was set to 1 m.

To mitigate the influence of boundary reflections on the results of the computational model, it is essential to implement an absorption boundary at the periphery of the fluid domain, a low-reflection boundary condition at the infinitely long end of the drill collar, and symmetrical boundaries for the remaining areas. During numerical simulation, a harmonic voltage signal of 1 V was applied to the inner arc of each circular disk of piezoelectric ceramic while the outer arc remained grounded. The radiated acoustic pressure *p*_0_ of the WD monopole acoustic source, driven by a single-frequency harmonic signal, was recorded at the spatial coordinates (1, 0, 0) m. By sweeping the frequency, the variation in the radiated acoustic pressure as a function of frequency was observed. The *S_v_* of the transducer was calculated using Equation (1).

#### 2.2.2. Transient Radiated Acoustic Pressure Waveform Calculation Methods

Downhole acoustic measurements require the use of transducers that can transmit sufficient acoustic energy into fluids to extend the detection range of AL tools. The electroacoustic conversion characteristics of transmitting transducers and excitation circuits determine the acoustic pressure waveforms emitted by the transducers. Studies on the transient response of transducers driven by different voltage excitation signals have shown that the maximum radiated power of the transducers is achieved when the width of the square pulse excitation signal is 0.5 times the resonant period [16,17]. For transducers used in ALWD tools, the electroacoustic conversion characteristics are complex because of the presence of drill collars and coupling materials. Consequently, it is necessary to investigate the radiated acoustic pressure signals of the monopole acoustic source during drilling, as driven by different excitation pulse voltage signals. In numerical simulations, transient dynamics analysis is frequently employed to obtain the radiated acoustic pressure waveform of a transducer driven by a time-varying excitation voltage signal [18]. This numerical calculation method requires constant changes in the excitation voltage signal and multiple repetitive numerical calculations, which require considerable computational resources and time.

**Figure 4:**
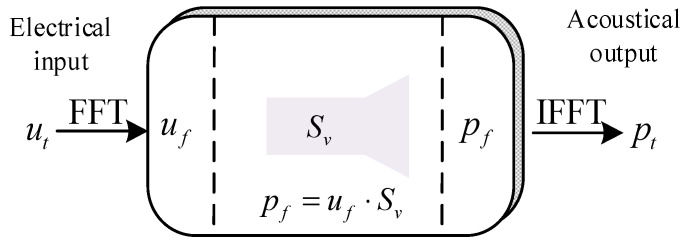
Schematic of the method used for calculating the transient response of transmitting transducers.

This paper proposes a computational method for calculating the transient radiated acoustic pressure signal of a transducer based on the complex transmitting voltage response. The proposed method can be employed to rapidly calculate the transient response of the transducer, as it only involves the fast Fourier transform (FFT). The fundamental premise of this methodology is that the transmitting transducers can be conceptualized as a linear, time-invariant system that modulates the complex amplitude of each frequency component of the input excitation voltage signal to generate an acoustic pressure signal output (Figure 4). This assumption is only applicable when the amplitude of the excitation voltage signal is small and the temperature and pressure are stable [19,20]. The frequency response of the system is defined as the complex transmitting voltage response (*S_v_*). The magnitude of the *S_v_* is referred to as the system gain, and the phase of the *S_v_* is designated as the phase shift of the system. Consequently, the far-field radiated acoustic pressure waveform of the transmitting transducer was calculated using the *S_v_*, which transformed the excitation voltage signal *u_t_* input to the system into an output acoustic pressure signal *p_t_*:

(5) $$p_{t}=IFFT\left(S_{v}\cdot FFT\left(u_{t}\right)\right),$$

where IFFT stands for inverse FFT.

The calculation method, based on the frequency-domain response of the transducer and the FFT, is applicable to the calculation of acoustic pressure waveforms and the calculation of the transient waveforms of the vibration velocity on the drill collar. A finite-element harmonic-response analysis can sufficiently determine the relation between the complex vibration velocity and the excitation voltage with frequency at the location under investigation. The FFT may be employed to obtain the transient velocity waveform of a sensor driven by an arbitrary voltage signal excited at any position on the drill collar.

**Figure 5:**
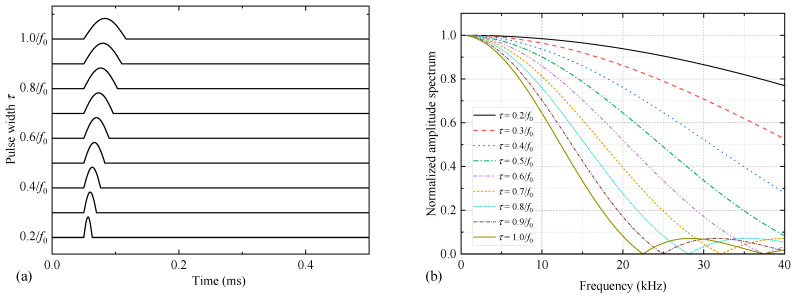
Time-domain waveform (**a**) and frequency spectrum (**b**) of half-sine excitation voltage signal with different pulse widths.

AL frequently employs a rectangular pulse excitation signal to stimulate the transducer, in conjunction with a transformer to augment the excitation power. This can generate high voltages in thousands of volts [21]. The rectangular pulse excitation signal with a pulse width of *τ* was transformed by the transformer into a sinusoidal signal with a similar half-period [22,23]. The time- and frequency-domain expressions for a half-sine pulse signal are as follows:

(6) $$u_{t}(t)=\left\{\begin{array}{c} \begin{array}{cc} U_{0}\sin\frac{\pi \left(t-t_{0}\right)}{\tau } & \left(t_{0}\leq t\leq t_{0}+\tau \right) \end{array}\\ \begin{array}{cc} 0 & \left(else\right) \end{array} \end{array},\right.$$

(7) $$u_{f}\left(\omega \right)=e^{-j\omega t_{0}}\frac{U_{0}}{2j}\left[F\left(\omega -\frac{\pi }{\tau }\right)-F\left(\omega +\frac{\pi }{\tau }\right)\right],$$

(8) $$F(\omega )=\frac{1}{j\omega }\left(1-e^{-j\omega \tau }\right),$$

where *U*_0_ is the maximum amplitude of the voltage signal, and *t*_0_ is the delay time of the voltage signal. Here, numerical simulations with *t*_0_ = 50 μs and *U*_0_ = 250 V were used. The time-domain waveform and frequency spectrum of the half-sine excitation voltage signal, as *τ* was varied from 0.2/*f*_0_ to 1.0/*f*_0_ are shown in Figure 5.

## 3. Results

Using the aforementioned methodology, the radiation characteristics of WD monopole acoustic sources with different mounting methods were simulated and analyzed.

### 3.1. Influence of Coupling Materials on Radiation Performance

#### 3.1.1. Different Natures of Coupling Materials

In the computational model shown in Figure 3, the coupling materials between the encapsulated piezoelectric vibrator and drill collar were assigned the following properties: fluid, polyetheretherketone (PEEK), and soft rubber. This allowed for an investigation into the impact of the coupling materials on the *S_v_* and the radiated acoustic pressure waveform of the WD monopole acoustic source. Table 3 presents the elastic parameters of the coupling materials. In the numerical simulation, the height of the coupling materials between the upper and lower bottom edges of the encapsulated arc-shaped piezoelectric vibrator and the boundary of the drill collar groove was designated as *h*_1_ = 4 mm. The thickness of the coupling materials between the inner vibrator and drill collar was defined as *d*_1_ = 2 mm. Furthermore, the influence of the protective cover on the radiation performance of the sound source was not considered.

**Figure 6:**
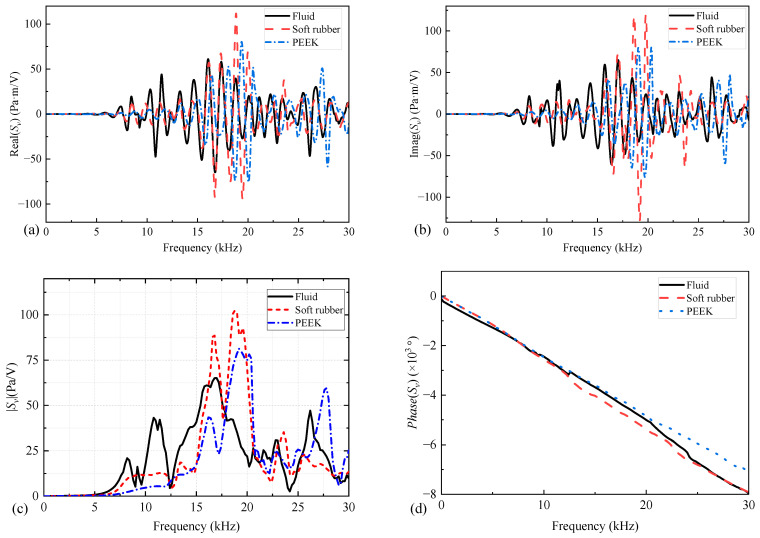
Real (**a**), imaginary (**b**), magnitude (**c**), and phase (**d**) versus frequency curves of the complex transmitting voltage response (*S_v_*) of the while-drilling monopole acoustic source when the coupling materials between the encapsulated piezoelectric vibrator and drill collar differed.

Figure 6 shows the complex transmitting voltage response versus the frequency of the WD monopole acoustic source when the nature of the coupling materials between the vibrator and drill collar differed. The complex transmitting voltage response magnitude versus frequency curve exhibited multiple peaks below 15 kHz when fluid was used as the coupling material. When the coupling material between the vibrator and drill collar was solid, the peak of the complex transmitting voltage response magnitude versus frequency curve shifted to high frequencies, and the bandwidth was narrower. Consequently, the effect of coupling materials on the acoustic properties of a WD monopole acoustic source cannot be discounted.

**Figure 7:**
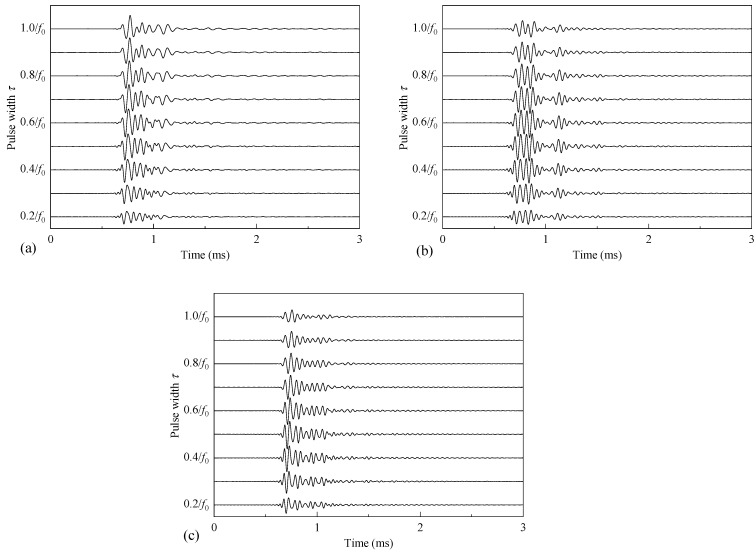
Acoustic pressure signals radiated by a while-drilling monopole acoustic source driven by half-sine excitation voltage signals with different pulse widths with coupling materials being a fluid (**a**), soft rubber (**b**), and PEEK (**c**).

**Figure 8:**
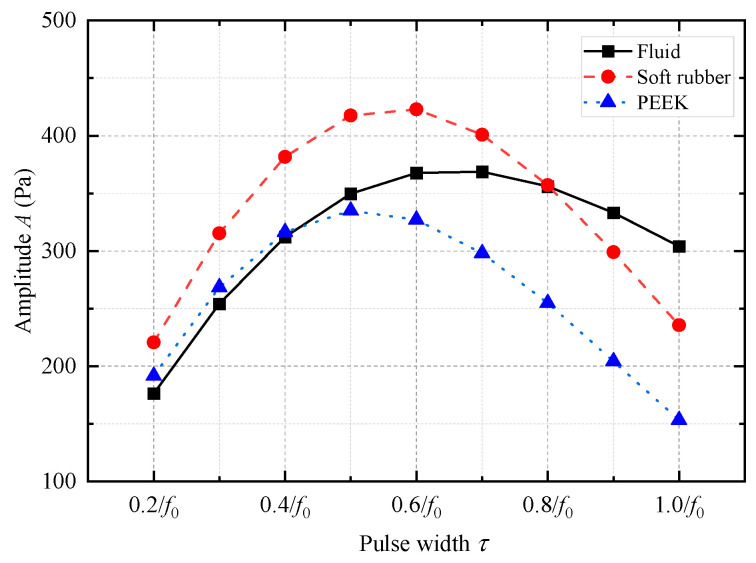
Variation in the amplitude of the acoustic pressure waveform radiated by the while-drilling monopole acoustic source with the excitation voltage pulse width, *τ*, when the coupling materials between the encapsulated piezoelectric vibrator and drill collar differed.

The acoustic pressure signals emitted by the WD monopole acoustic source are related to the transmitting voltage response and excitation voltage signal of the source. Using Equation (5), the acoustic pressure signals radiated by the WD monopole acoustic source with different coupling materials under the excitation of half-sine voltage signals with different pulse widths were calculated, and the results are shown in Figure 7. The radiated acoustic pressure waveforms generated by the WD monopole acoustic source with different coupling materials exhibited considerable morphological differences. The waveforms of the radiated acoustic pressure waveforms from the WD monopole acoustic source were similar to Ricker signals with a longer tail when fluid was used as the coupling material. However, when soft rubber was used as the coupling material, the waveforms of the radiated acoustic pressure from the WD monopole acoustic source resembled four-period cosine envelope signals. Figure 8 shows the variation curve of the radiated acoustic pressure waveform amplitude *A* of the WD monopole acoustic source as a function of the half-sine excitation pulse width *τ*. The waveform amplitude *A* was defined as follows:

(9) $$A={\left[\sum_{t=T_{1}}^{T_{2}}p^{2}(t)/N\right]}^{\frac{1}{2}},$$

where *T*_1_ and *T*_2_ are the start and end moments of the waveform window, respectively, and *N* is the number of waveform sample points in the time window. The range of the acoustic pressure waveform window during the calculation was 0~2 ms. As shown in Figure 8, the amplitude of the acoustic pressure signals emitted by the monopole acoustic source tended to first increase and then decrease with an increase in the pulse width. The amplitude of the radiated acoustic pressure waveform of the WD monopole acoustic source with coupling materials of fluid, soft rubber, and PEEK reached its maximum when the pulse width *τ* of the half-sine excitation signal was 0.7/*f*_0_, 0.6/*f*_0_, and 0.5/*f*_0_, respectively.

The practical application of the acoustic performance of the source needs to meet certain requirements. On the one hand, the expectation of the radiated acoustic pressure amplitude of the WD monopole acoustic source is large, to improve the measurement results of the depth of detection and signal-to-noise ratio. On the other hand, the frequency of the acoustic pressure waveform should not be excessively high or low. An excessively low frequency may not meet the requisite measurement resolution, whereas an excessively high frequency is susceptible to attenuation. Furthermore, it has been demonstrated that the longitudinal vibration generated by a WD monopole acoustic source along the length of the drill collar will not enhance the energy of the formation wave. Instead, it will generate a strong collar wave, which will interfere with the processing of the ALWD waveform [15,24]. The transducer-mounting method can affect the amplitude and frequency characteristics of the radiated acoustic pressure waveform of the WD monopole acoustic source, as well as the amplitude of the axial vibration of the drill collar. Thus, when analyzing the acoustic performance of the WD monopole acoustic source, it is necessary to consider the radiation characteristics of the transducer and the strength of the axial vibration on the surface of the drill collar.

**Figure 9:**
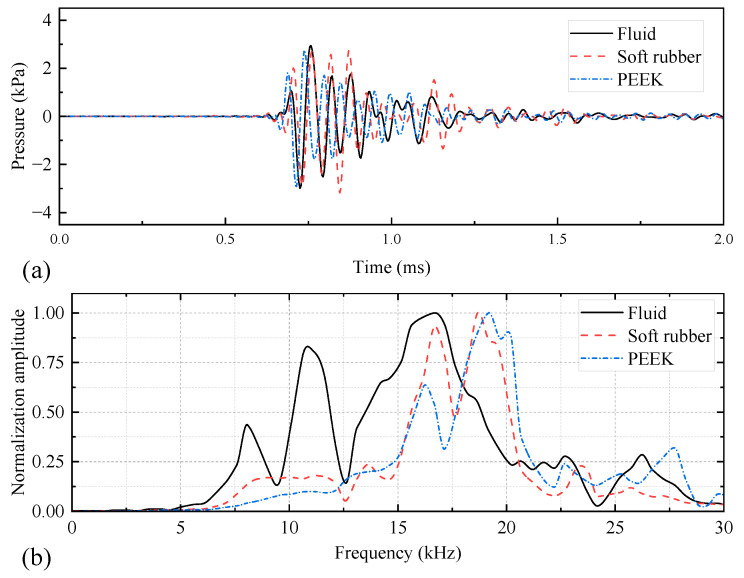
Waveforms (**a**) and spectra (**b**) of acoustic pressure radiated at (1, 0, 0) m from while-drilling monopole acoustic sources with different coupling materials, driven by a voltage signal with a pulse width of 0.6/*f*_0_.

**Figure 10:**
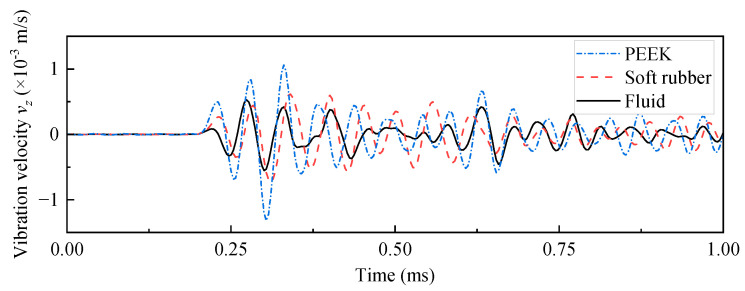
Waveforms of axial vibration velocity at the surface of the drill collar (0.1, 0, 0.9) m for while-drilling monopole acoustic source with different coupling materials, driven by a voltage signal with a pulse width of 0.6/*f*_0_.

**Figure 11:**
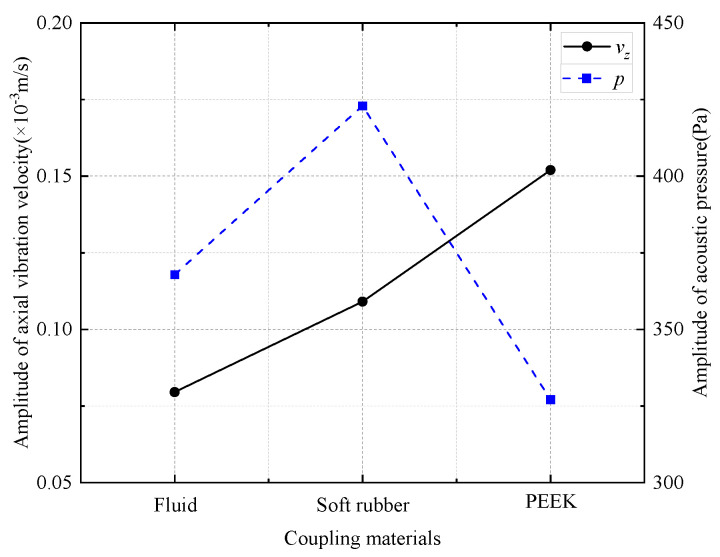
Curves of radiated acoustic pressure amplitude and axial vibration velocity waveform amplitude for different coupling materials.

The acoustic performance of the WD monopole acoustic source is influenced by differences in the coupling materials between the encapsulated piezoelectric vibrator and drill collar. Figure 9 shows the acoustic pressure waveforms and normalized spectra recorded at (1, 0, 0) m spatial coordinates for the WD monopole acoustic source with different coupling materials between the transducer and drill collar, driven by a half-sine excitation pulse with a pulse width of 0.6/*f*_0_. As shown in Figure 9a, the acoustic pressure waveforms emitted by the WD monopole acoustic source with different coupling materials exhibited distinct trailing signals. The presence of the trailing signals may potentially interfere with the processing of stratigraphic data [25]. Therefore, further investigation into the transient suppression of WD monopole acoustic sources is required, although this is not the focus of the present paper. Figure 9b shows that the normalized spectrum of the radiated acoustic pressure waveform of the WD monopole acoustic source when coupled with PEEK, exhibited an increased prevalence of high-frequency components above 17.5 kHz. Thus, this configuration was unsuitable as a WD monopole acoustic source. Figure 10 shows the axial vibration velocity waveforms of WD monopole acoustic sources with different coupling materials, driven by a half-sine excitation pulse with a pulse width of 0.6/*f*_0_, received at a source distance of 0.9 m on the drill collar surface. Figure 11 presents a comparison of the magnitude of the amplitude of the acoustic pressure signals generated by WD monopole acoustic sources with different coupling materials and the magnitude of the axial vibration velocity waveforms received on the surface of the drill collar. The waveform window ranged from 0 to 2 ms for acoustic pressure amplitude calculations and from 0 to 1 ms for vibration velocity amplitude calculations. As illustrated in Figure 11, the excitation of the drill collar axial vibration by a WD monopole acoustic source was less pronounced. In addition, the amplitude of the radiated acoustic pressure waveform was greatest when soft rubber was employed as the coupling material compared with PEEK. Thus, the selection of coupling materials for soft rubber is optimal.

**Figure 12:**
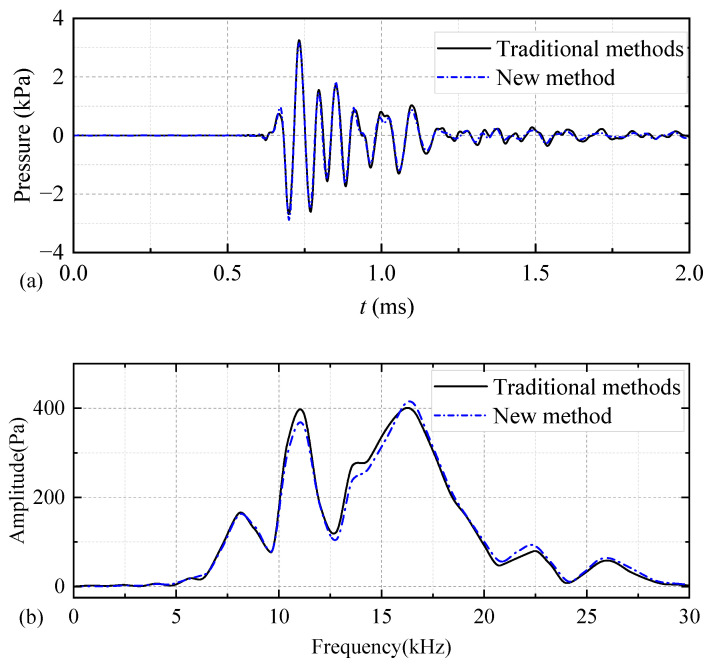
Waveforms (**a**) and spectra (**b**) of acoustic pressure generated by a half-sine excitation voltage signal with a pulse width of 0.7/*f*_0_ exciting a while-drilling monopole acoustic source at the spatial coordinates (1, 0, 0) m (with fluid as a coupling material) computed using two different methods.

To validate the proposed methodology, based on the complex transmitting voltage response, the present results were compared with those obtained via the traditional method (finite-element transient analysis). The computational model for the finite-element transient analysis was identical to that employed for the harmonic-response analysis illustrated in Figure 3, except that the harmonic-response analysis was transformed into a transient analysis. This involves modifying the harmonic voltage of 1 V applied to the inner arc of the piezoelectric ceramic wafer to a half-sine voltage signal with a pulse width of 0.7/*f*_0_. Using the fluid as the coupling material between the encapsulated piezoelectric vibrator and drill collar, two distinct numerical calculation methods were employed. This was to obtain the acoustic pressure waveforms and corresponding spectra generated by the WD monopole acoustic source at the spatial coordinates of the (1, 0, 0) m position. The calculation results are shown in Figure 12. The results of both methods were essentially identical, thereby confirming the accuracy of the methodology employed in this study. The method of calculating the transient response of the transducer based on the complex transmitting voltage response (new methods) was divided into two steps: finite element harmonic response analysis and IFFT. The finite element harmonic response sweep calculation time was 3 h, and the time consumed by IFFT was negligible; hence, the calculation results shown in Figure 7a were obtained in 3 h in total. In the case of unchanged computational conditions, the method of the finite-element transient analysis (traditional method) took a time of 2.6 h for a single calculation, and the calculation results shown in Figure 7a were obtained in 49.4 h in total. Compared with finite-element transient analysis, the proposed methodology exhibited a higher calculation speed, which was conducive to the expeditious design of the excitation voltage signal.

#### 3.1.2. Change in Thickness of Coupling Materials

**Figure 13:**
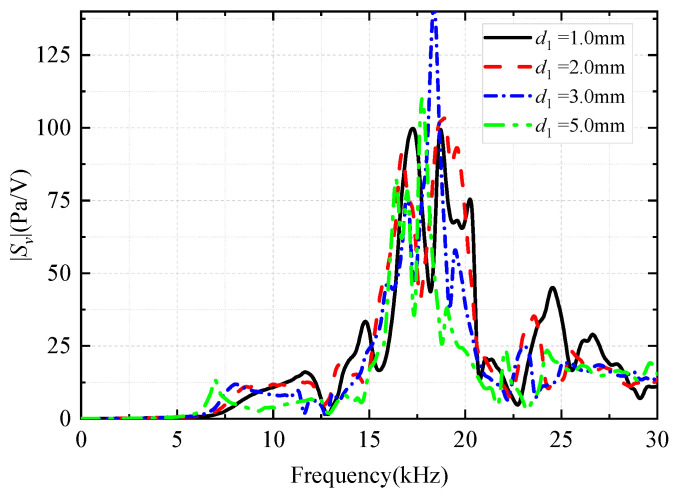
Effect of the thickness of coupling materials on the magnitude versus frequency curve of the transmitting voltage response *S*_v_ of the while-drilling monopole acoustic source.

The thickness (*d*_1_) of the coupling materials between the inner arc surface of the encapsulated arc-shaped piezoelectric vibrator and the drill collar was changed (the depth of the grooves in the drill collar was changed). The coupling material (soft rubber) between the transducer and drill collar (Figure 3) remained unchanged. The height of the coupling materials, *h*_1_, was 4 mm, and the structure of the protective cover was not considered yet. This allowed for an analysis of the effect of the thickness of the coupling materials, *d*_1_, on the radiation performance of the WD monopole acoustic source. Figure 13 shows the variation in the transmitting voltage response of the WD monopole acoustic source with frequency as the thickness of the coupling materials was altered. With an increase in the thickness of the coupling materials, the spectral peaks of the transmitting voltage response of the WD monopole acoustic source initially rose and then declined, with the position of the spectral peaks gradually shifting toward low frequencies.

**Figure 14:**
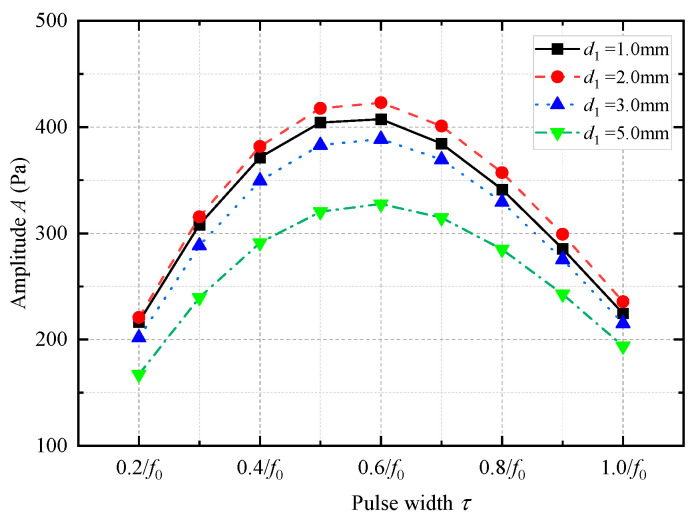
Variation in the amplitude of the radiated acoustic pressure waveform of the while-drilling monopole acoustic source with the pulse width of the excitation voltage signal *τ* with respect to changes in the thickness of the coupling material (soft rubber).

**Figure 15:**
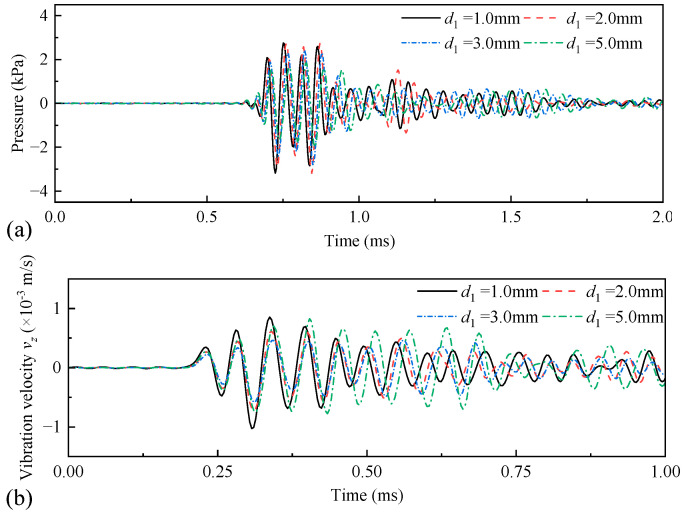
Radiated acoustic pressure waveforms (**a**) recorded at spatial coordinates of (1, 0, 0) m, and axial vibration velocity waveforms (**b**) recorded at coordinates of (0.1, 0, 0.9) m on the drill-collar surface when the thicknesses of the coupling material, *d*_1_, varied.

**Figure 16:**
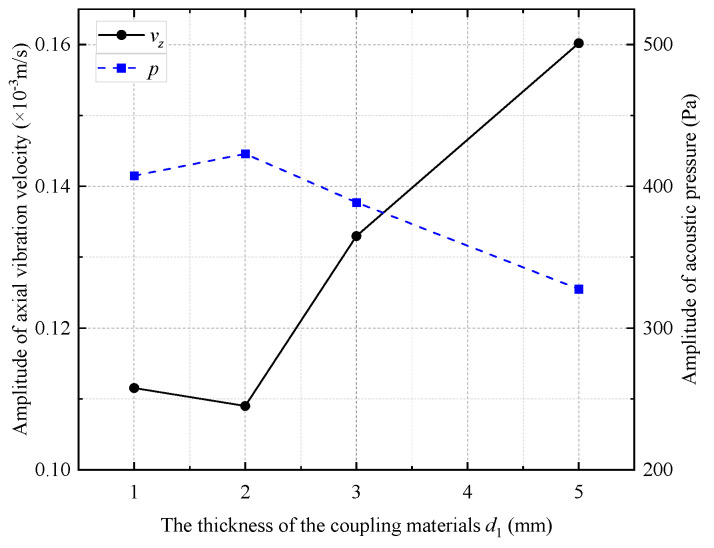
Curves of radiated acoustic pressure amplitude and axial vibration velocity waveform amplitude with the thickness of coupling materials.

The amplitude of the radiated acoustic pressure waveform produced by the WD monopole acoustic source with different thicknesses of coupling materials varied with the pulse width of the half-sine excitation voltage signal (Figure 14). The amplitude of the radiated acoustic pressure waveform was at its maximum when the pulse width of the half-sine voltage excitation voltage was 0.6/*f*_0_. This was despite the variation in the thickness of the soft rubber. Figure 15 shows the radiated acoustic pressure waveforms of the WD monopole acoustic source and the axial vibration waveforms recorded on the surface of the drill collar for a half-sinusoidal voltage excitation with a pulse width of 0.6/*f*_0_. Figure 16 shows a statistical analysis of the variation in the amplitude of the radiated acoustic pressure waveform and the amplitude of the axial vibration velocity waveform of the drill collar with the thickness of the coupling materials. The optimal thickness of the coupling material between the transducer and drill collar was 2 mm when soft rubber was used. This configuration resulted in a larger amplitude of the radiated acoustic pressure waveform and a smaller amplitude of the axial vibration velocity waveform of the drill collar.

#### 3.1.3. Change in Height of Coupling Materials

**Figure 17:**
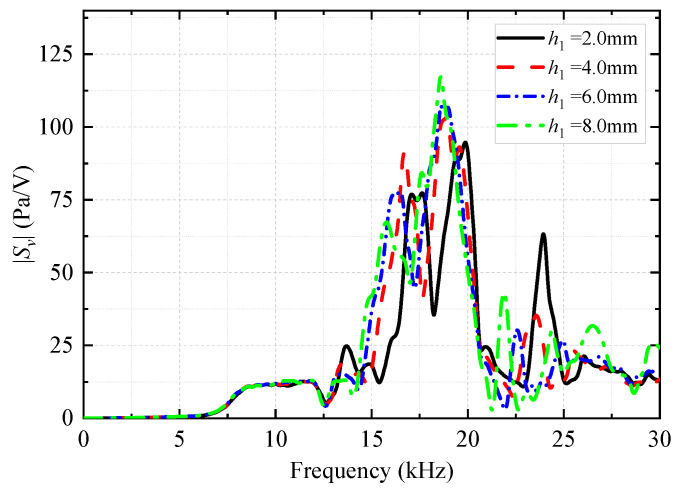
Effect of the heights of coupling materials on the magnitude versus frequency curve of the transmitting voltage response *S*_v_ of the while-drilling monopole acoustic source.

The height (*h*_1_) of the coupling materials between the upper and lower bottom surfaces of the encapsulated vibrator and drill collar was changed (the height of the grooves of the drill collar was changed). The coupling material (soft rubber) between the transducer and drill collar in Figure 3 remained unchanged. The effect of the height *h*_1_ of the coupling materials on the radiation performance of the WD monopole acoustic source was analyzed. Figure 17 shows the variation in the transmitting voltage response of the WD monopole acoustic source with frequency at varying heights of the coupling materials. With an increase in the height of the coupling materials, the peak amplitude of the transmitting voltage response gradually increased, and the peak of the spectrum gradually shifted toward low frequencies.

**Figure 18:**
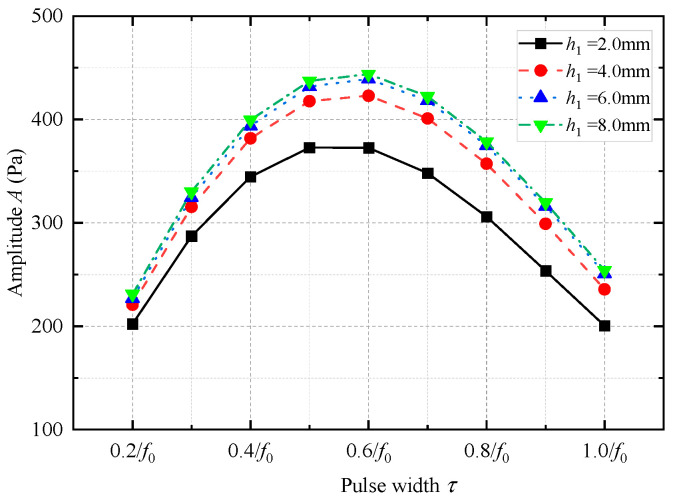
Variation in the amplitude of the radiated acoustic pressure waveform of the while-drilling monopole acoustic source with the pulse width of the excitation voltage signal *τ* with respect to changes in the height of the coupling material (soft rubber).

**Figure 19:**
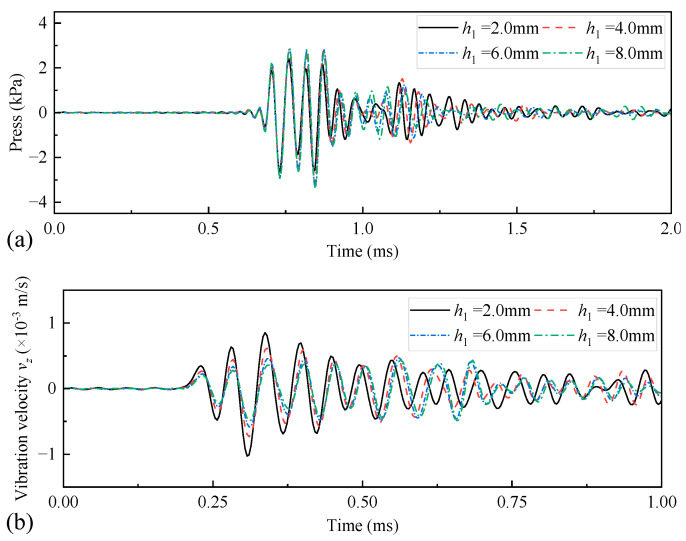
Radiated acoustic pressure waveforms (**a**) recorded at spatial coordinates of (1, 0, 0) m, and axial vibration velocity waveforms (**b**) recorded at coordinates of (0.1, 0, 0.9) m on the drill-collar surface when the height of the coupling material *h*_1_ varied.

**Figure 20:**
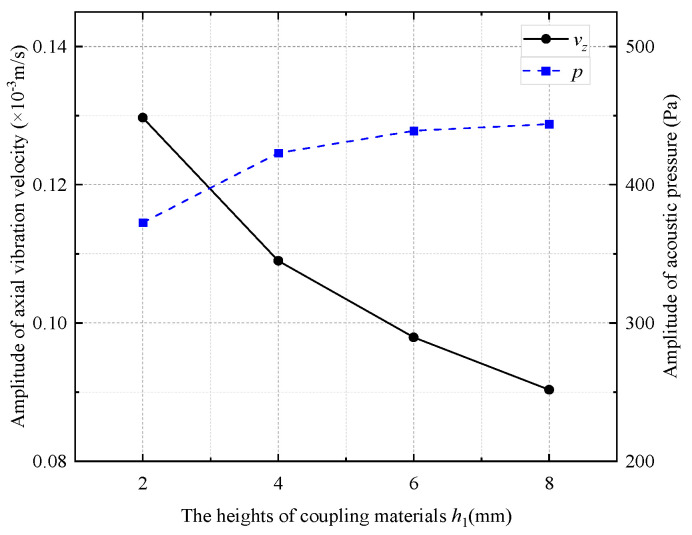
Curves of radiated acoustic pressure amplitude and axial vibration velocity waveform amplitude with the heights of coupling materials.

Figure 18 shows the amplitude of the radiated acoustic pressure waveform generated by the WD monopole acoustic source when the height of the coupling materials was varied with the pulse width of the half-sine excitation voltage signal. The amplitude of the radiated acoustic pressure waveforms was largest when the pulse width of the half-sine excitation voltage was 0.6/*f*_0_. Figure 19 shows the radiated acoustic pressure waveforms of the WD monopole acoustic source and the axial vibration waveforms recorded on the surface of the drill collar when the pulse width of the half-sine voltage excitation was 0.6/*f*_0_. Figure 20 counts the amplitude of the radiated acoustic pressure waveforms and the amplitude of the axial vibration waveforms of the drill collar. When soft rubber was used as the coupling material, the higher the coupling material, the greater the amplitude of the radiated acoustic pressure waveforms and the weaker the axial vibration recorded on the drill collar surface.

### 3.2. Influence of Protective Cover on Radiation Performance

**Figure 21:**
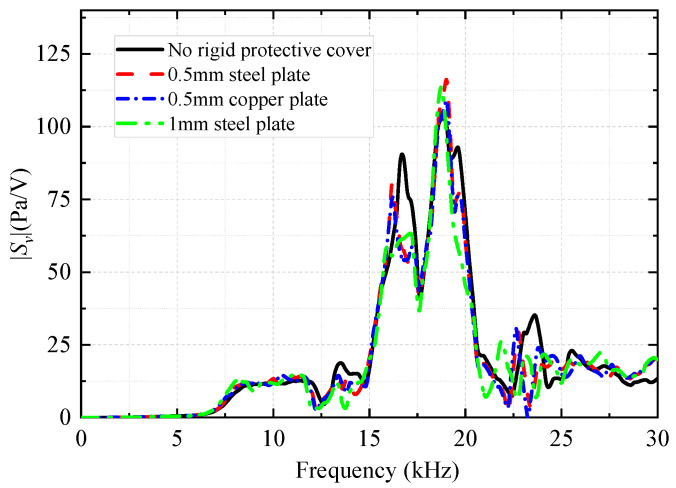
Effect of the thickness and material of the metal plate used as a protective cover on the magnitude versus frequency curve of the transmitting voltage response *S_v_* of the while-drilling monopole acoustic source.

Once the transducer is mounted onto the drill collar, the transducer must be protected from the drilling fluid by fitting a protective sheet metal cover on the outermost side. In the harmonic-response analysis of the WD monopole acoustic source with different thicknesses and materials of the metal plate as the protective cover, the coupling material (soft rubber) between the transducer and the drill collar remained unchanged. The thickness of the coupling materials was set to *d*_1_ = 2 mm, and the height of the coupling materials was set to *h*_1_ = 4 mm. Figure 21 shows the variation in the transmitting voltage response quantities with frequency for the WD monopole acoustic source with different protective covers.

**Figure 22:**
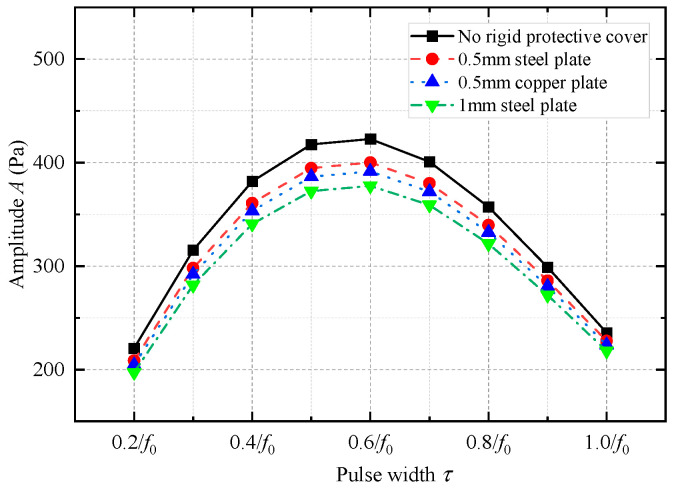
Variation in the amplitude of the acoustic pressure waveform emitted by a while-drilling monopole acoustic source with the pulse width of the excitation voltage signal *τ* when metal plates of different thicknesses and materials were used as protective covers.

**Figure 23:**
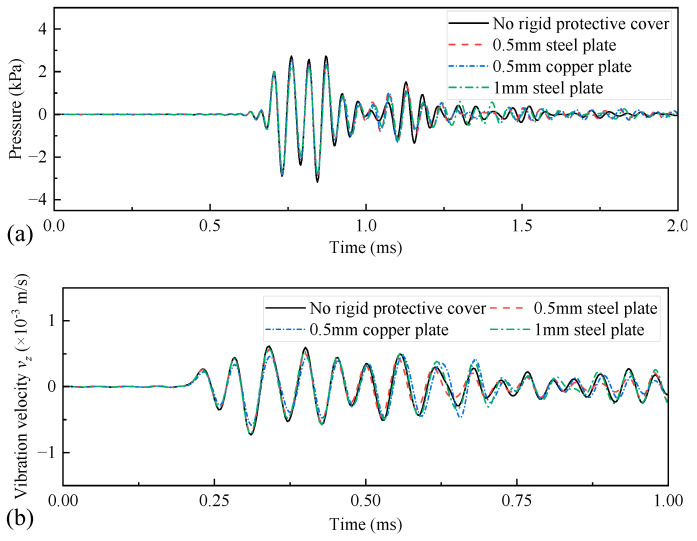
Radiated acoustic pressure waveforms (**a**) recorded at spatial coordinates of (1, 0, 0) m, and axial vibration velocity waveforms (**b**) recorded at coordinates of (0.1, 0, 0.9) m on the drill-collar surface when metal plates of different thicknesses and materials were used as protective covers.

**Figure 24:**
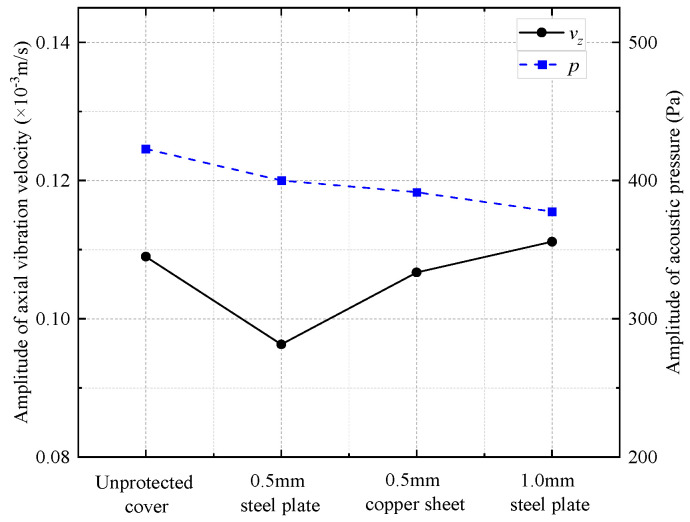
Curves of radiated acoustic pressure amplitude and axial vibration velocity waveform amplitude with the material and thickness of the protective cover.

In numerical simulations, the elastic parameters of steel and drill collar materials are identical. The density and longitudinal and transverse wave speeds of copper are 8900 kg/m^3^, 4710 m/s, and 2260 m/s, respectively. As shown in Figure 21, when the metal plate was thin, the shield did not substantially affect the transmitting voltage response, and most of the frequency components of the acoustic energy radiated through the shield into the infinite fluid. Figure 22 shows the amplitude of the radiated acoustic pressure waveform of the WD monopole acoustic source driven by half-sine excitation signals with different pulse widths. The protective cover slightly reduced the amplitude of the radiated acoustic pressure waveform of the WD monopole acoustic source. Furthermore, the thinner the protective cover, the lower the effect on the electroacoustic conversion characteristics of the WD monopole acoustic source, and vice versa. Figure 23 shows the radiated acoustic pressure waveforms of WD monopole acoustic sources with different protective shield structures driven by half-sine excitation pulses with a pulse width of 0.6/*f*_0_ and the axial vibration waveforms excited on the drill collar surface. After counting the waveform amplitude using Equation (9), Figure 24 shows the influence of the material and thickness of the protective cover on the radiation performance of the WD monopole acoustic source. The choice of a steel plate (thickness = 0.5 mm) was the most suitable protective cover.

## 4. Conclusions

The acoustic pressure signal radiated by the WD monopole acoustic source is dependent on the electroacoustic conversion characteristics of the transducer and excitation voltage signal. To improve the acoustic pressure energy radiated by the WD monopole acoustic source, this study numerically investigated the influence of the transducer-mounting method on the radiation performance of the WD monopole acoustic source using multiphysical field FEM. Furthermore, we proposed a numerical calculation method that can rapidly calculate the transient response of the transducer when the WD monopole acoustic source is driven by the voltage excitation. The waveforms of the radiated acoustic pressure signals generated by the WD monopole acoustic source driven by half-sine excitation voltage signals with different pulse widths were calculated using the proposed method.

The numerical simulation results showed that when the encapsulated vibrator was mounted on the drill collar, it was appropriate to use soft rubber with a thickness of 2 mm and a large height as the coupling material and 0.5-mm thick steel for the protective cover. In this case, the WD monopole acoustic source can radiate high acoustic energy into the fluid, and the axial vibration on the surface of the drill collar is weak. As the pulse width of the WD monopole acoustic source increased, the amplitude of the radiated acoustic pressure signal increased and then decreased, and there was always a large value obtained. The WD monopole acoustic source with soft rubber as a coupling material radiated the acoustic pressure waveform with the maximum energy amplitude when the pulse width of the half-sine excitation signal was 0.6/*f*_0_. Thus, a suitable transducer-mounting method and pulse width of the excitation voltage signal are required to increase the radiation performance of the WD monopole acoustic source.

Once the transmitting voltage response of the transducer was obtained, the proposed calculation method was employed to quickly calculate the acoustic wave signal radiated by the WD monopole acoustic source mounted using different methods under an arbitrary excitation voltage signal. The numerical simulation results provide valuable guidance for selecting the transducer mounting method and designing the excitation voltage signal during ALWD. Furthermore, the numerical simulation method proposed herein can be used to analyze the electroacoustic conversion characteristics of most complex transducers.

## Figures and Tables

**Table 1:** Elastic parameters and structural dimensions of media in the computational model.

Type	*V_p_* (m/s)	*V_s_* (m/s)	*ρ* (kg/m^3^)	*R* (m)
Fluid inside drill collar	1500	0	1000	0.04
Drill collar	5900	3230	7800	0.1
Fluid outside drill collar	1500	0	1000	Boundless
Epoxy resin	2757.6	1306.7	1320.6	-

**Table 2:** Parameters of PZT-5G material.

Type	PZT-5G	Unit
Densities	7400	kg/m^3^
Flexibility constant *S*^E^	{1.51 × 10^−11^, −5.8 × 10^−12^, 1.5 × 10^−11^, −4.82 × 10^−12^, −4.82 × 10^−12^, 2.83 × 10^−11^, 0, 0, 0, 4.3 × 10^−11^, 0, 0, 0, 0, 4.3 × 10^−11^, 0, 0, 0, 0, 0, 4.18 × 10^−11^}	1/Pa
Piezoelectric constant *d*	{0, 0, −1.86 × 10^−10^, 0, 0, −1.86 × 10^−10^, 0, 0, 4.20 × 10^−10^, 0, 6.6 × 10^−10^, 0, 6.6 × 10^−10^, 0, 0, 0, 0, 0}	C/N
Dielectric constant *ε*	{2404.5, 2404.5, 2000}	1

**Table 3:** Elastic parameters of coupling materials between encapsulated piezoelectric vibrator and drill collar.

Type	*V_p_* (m/s)	*V_s_* (m/s)	*ρ* (kg/m^3^)
Fluid	1500	0	1000
PEEK	2300	940	1200
Rubber	1100	500	1100

## Data Availability

Data will be made available on request.
